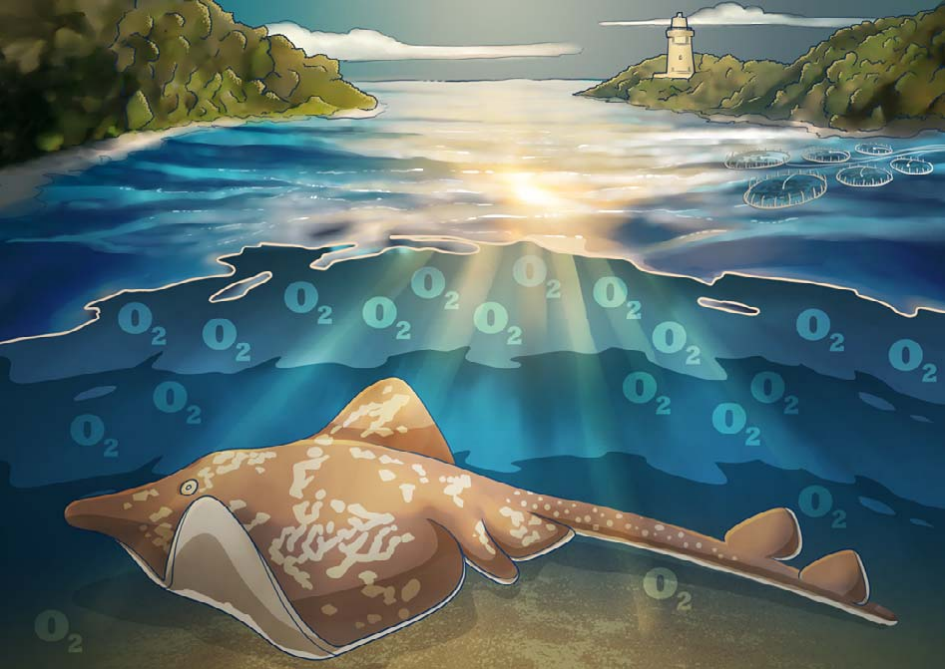# He was a skater boy, but will the Maugean skate(r) conform or rebel?

**DOI:** 10.1093/conphys/coaa043

**Published:** 2020-07-03

**Authors:** Phillipa K Beale

**Affiliations:** Australian National University, 134 Linnaeus Way, Acton, ACT 2601, Australia

We know surprisingly little about the endangered and endemic Maugean Skate (*Zearaja maugeana*). Despite its reputation as a rebel, it turns out this animal is a bit of a conformer, an oxyconformer to be exact. That means that this skate tolerates low-oxygen environments by changing how its cells utilize energy.

There are two options for animals to live happily in low-oxygen environments. They can either regulate or conform. Some animals regulate their physiology to be better at taking up oxygen when levels get low, while other animals conform their needs to the amount of oxygen in the environment. Those animals can do this either by switching to anaerobic metabolism (making energy without oxygen) or by lowering their metabolic rates and therefore their energy requirements. The last option is the most common among low-oxygen-tolerating animals.

The water in the Macquarie Harbour, where the Maugean skate hangs out, is layered. The top layer is warm, fresh water and the bottom layer is cold, salt water. The middle layer is mixed. As Morash et al. (2020) found out, the amount of dissolved oxygen at the skates’ preferred depths ranges from 90% saturation down to only 30% saturation; although, there are even areas with essentially no oxygen. Morash and her team wanted to know how the Maugean skate manages to swim around through all those different oxygen environments and how well it copes with even lower oxygen levels.

The team collected skates from the Harbour, transported them to the laboratory and then, for 48 hours, maintained half of the animals at oxygen levels of 20% saturation and the other half at oxygen levels of 50% saturation. Then, the team looked for differences in blood parameters that could indicate how well skates were taking up oxygen from the environment. The team also took measurements to estimate the skates’ metabolic rates across a range of oxygen levels. Was it possible that the skates were using anaerobic metabolism? To find out, the team measured glycogen, lactate and other metabolic and stress markers.

The results indicated that the Maugean skate was not particularly worried about low oxygen levels. Based on the blood parameters and metabolic rate data, the skates did not seem to be regulating oxygen uptake or adjusting their energy requirements. Rather, the skates were relying a bit more on anaerobic metabolism, as indicated by lower glycogen and higher lactate levels. However, they were not exhibiting a stress response. The team found this really interesting and now wonder how long these skates can sustain anaerobic metabolism.

The oxygen levels in Macquarie Harbour are continuing to decline, most likely due to salmon farming, development and other human activities. What would happen if the oxygen levels in the Harbour got really low? The Maugean skate could swim up through the layers in the water to higher oxygen levels, but then they would encounter warm, freshwater, which may pose other problems. With this kind of information, we can focus conservation efforts on the things that matter most to the Maugean skate, like preventing this cool conformer from skating out of existence!

**Illustrations**: Erin Walsh, ewalsh.sci@gmail.com